# Pax6 as a Therapeutic Target: Convergent Molecular Pathways in the Regulation of Aniridia, Neurogenesis, Neurodegeneration and Oncology

**DOI:** 10.3390/cells15040324

**Published:** 2026-02-10

**Authors:** Marina Skorkina, Tatiana Kovaleva, Irina Predeina, Nikita Smirnov, Irina Mukhina

**Affiliations:** Institute of Fundamental Medicine, Privolzhsky Research Medical University, 10/1 Minin Sq., Nizhniy Novgorod 603005, Russia; predeina_iv@pimunn.net (I.P.); smirnov_na@pimunn.net (N.S.); muhina_i@pimunn.net (I.M.)

**Keywords:** Pax6, cell cycle, proliferation, neurogenesis, aniridia, neurodegeneration, oncology

## Abstract

**Highlights:**

**What are the main findings?**

- Pax6 functions as a pivotal transcription factor that controls the balance between cell proliferation, differentiation, and survival, thereby influencing tissue homeostasis in both health and disease.
- Pax6 can behave as either a tumor inhibitor or an oncogene during tumor development, demonstrating a context-dependent dual function.

**What are the implications of the main findings?**

- Pax6 may represent a viable target in selected pathophysiological settings.
- Future investigations should prioritize understanding the precise regulation of Pax6 isoforms, and developing isoform-specific inhibitors tailored to the predominant form driving a given disease, such as aniridia, specific cancers, or metabolic disorders.

**Abstract:**

Pax6 is a multifunctional transcription factor that orchestrates cell cycle progression at distinct stages of early embryonic neurogenesis and serves as a molecular mediator integrating multiple signaling pathways associated with pathological processes. Within this framework, Pax6 is regarded as an attractive molecular target for developing new drugs aimed at combating neurodegeneration, oncology, and aniridia. The present review aims to examine published studies describing various Pax6-dependent molecular pathways to identify common principles and condition-specific differences in Pax6-regulated cascades in health and disease. These insights may contribute to the conceptual foundation for developing new therapeutic strategies targeting Pax6 as a molecular regulator. This review summarizes the data demonstrating a central role of Pax6 in governing the neuronal cell cycle in health and pathology. It is possible that Pax6 may act as a therapeutic target in certain pathophysiological conditions; however, the effectiveness of such a strategy will depend on the substrate chain in the signaling pathway, its branching, and the redundancy of mediators involved.

## 1. Introduction

Pax6 is a pleiotropic factor [1] and is considered a master gene in the control of eye morphogenesis and evolution [2,3]; however, its function is not limited to vision. Pax6 is critically involved in shaping the central nervous system (CNS) [4], as well as other organs, including the pancreas, pituitary gland and nose [5,6]. Pax6 controls neurogenesis and gliogenesis [7,8], and contributes to the establishment of boundaries between emerging regions of the forebrain [9]. Acting as a molecular switch, Pax6 controls the specification and terminal differentiation of different cell types. The production of Pax6 protein is tightly regulated at both the pre-mRNA and post-transcriptional levels [10,11].

The role of Pax6 as a dual transcriptional activator and repressor is known [12,13,14]. During embryonic development, Pax6 engages two separate DNA-binding domains alongside a proline-serine-threonine–rich activation domain to control transcription of downstream targets [15,16,17]. Its regulatory influence is further mediated through the recruitment of chromatin-remodeling complexes, which facilitate gene expression by loosening heterochromatic regions [18,19]. Pax6 has been shown to influence the morphology and electrophysiological properties of neurons in the prethalamic neuroepithelium [20].

This review aims to provide a comprehensive synthesis of studies describing various molecular signaling pathways involving Pax6, to find similarities and differences in the signaling cascades that Pax6 can activate in health and disease, which will allow these data to be used to search for new therapeutic strategies targeting Pax6 as a molecular target. The study of Pax6 gene role included the following criteria: assessment of the gene’s regulatory role in proliferation, differentiation, and maturation of ectoderm-derived cells during development; study of its transcriptional network and downstream signaling targets; Pax6 involvement in cellular metabolism; and its association with aniridia, neurodegenerative disorders, and cancer.

## 2. Regulatory Role of Pax6 in Embryogenesis

### 2.1. Molecular Organization of the Pax6 Gene, Its Isoforms and Functions

The Pax6 gene encodes a tissue-specific transcriptional factor located on the short arm of chromosome 11 (locus 11p13) [21] and is highly conserved across vertebrate and invertebrate species. The paired box (Pax) gene family was initially identified in Drosophila as segmentation genes, including paired (prd), gooseberry-distal (gsb-d), and gooseberry-proximal (gsb-p), and subsequently in other animal species. Comparative analyses have revealed nine Pax genes in both mice (Pax1–9) and humans (PAX1–9), all of which contain a conserved 128-amino acid N-terminal paired domain (PD). This domain may occur alone (Pax1, Pax9), with a full-length homeodomain (Pax3, Pax4, Pax6, Pax7), or in a truncated form (Pax2, Pax5, Pax8) [22].

The structure of the gene and its encoded protein have been studied in detail and described in a number of works [1,23,24,25,26]. Pax6 consists of 14 exons, its expression is controlled by three independent promoters (P0, P1, and Pα) [27], which produce a number of alternative transcripts [28]. Analysis of the Pax6 promoter revealed a TATA-like motif at –26 bp, two CCAAT boxes at positions −70 and −100 bp, and a 38 bp poly(CA) sequence located 992 bp upstream of the initiation site [22].

Expression of Pax6 is spatially restricted to regions of the optic cup, forebrain, spinal cord, hindbrain, nasal epithelium, and lens placode [22]. Pax6 is essential for CNS development, eye morphogenesis, lens induction, and the formation and function of endocrine cells [29]. It also maintains progenitor cell pools in the cerebral cortex and spinal cord and preserves the multipotency of retinal progenitors [10]. Heterozygous mutations of Pax6 lead to eye malformations in humans (aniridia), mice (small eye phenotypes), and Drosophila (absence of eyes) [22].

More than 700 pathogenic variants of Pax6 have been identified to date. Many of these generate premature stop codons through nonsense mutations, frameshifts, or splicing defects, resulting in haploinsufficiency. Missense mutations predominantly affect the paired domain and, less frequently, the homeodomain. Splicing mutations account for up to 30% of pathogenic variants, occurring at canonical 5′ and 3′ splice sites, as well as in noncanonical splice sites, regulatory sequences, deep intronic regions, and untranslated regions (UTRs) [28].

The Pax6 protein is tightly regulated at pre-mRNA, post-transcriptional, and protein levels [30]. Levels of Pax6 that are too low or too high can severely disrupt tissue development and homeostasis. The molecular mechanisms regulating Pax6 expression are still unclear. MicroRNA-mediated post-transcriptional regulation represents a key mechanism for maintaining Pax6 levels [10,30].

**Figure 1:**
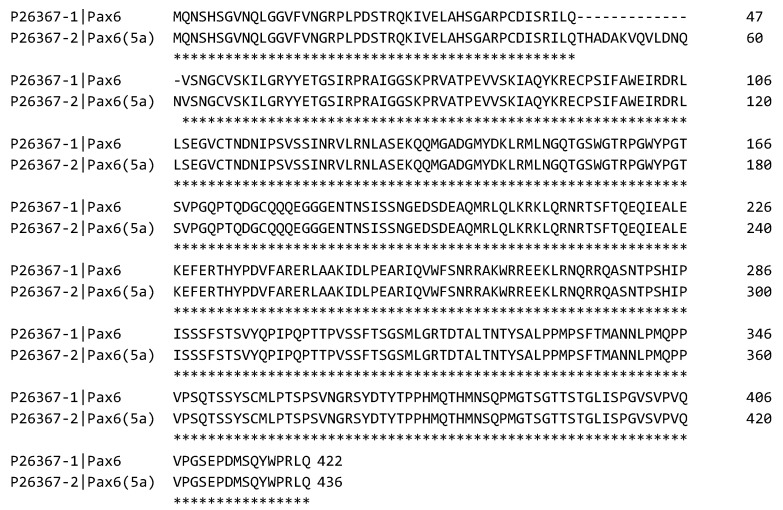
A comparative analysis of the amino acid sequences of the Pax6 isoforms, canonical Pax6 and Pax6(5a) [42,43,44]. Asterisks (*) indicate amino acid residues conserved between the aligned sequences, whereas dashes (-) denote gaps introduced during the alignment to accommodate insertions or deletions.

In mammals, Pax6 gives rise to three isoforms: canonical Pax6, Pax6(5a), and Pax6ΔPD (Figure 1). The canonical protein consists of 422 amino acids, featuring an N-terminal paired domain (PD, 128 residues), a paired homeodomain (HD, 61 residues) connected by a flexible linker, and a C-terminal proline-serine-threonine-rich (PST) domain (152 residues) [28]. PD and HD mediate DNA binding, whereas PST provides transactivation capacity [16,31,32,33]. The PD comprises two subdomains, PAI and RED, each with a conserved helix-turn-helix motif for DNA interaction, while the HD contains three α-helices with distinct DNA-binding specificity. A 72-amino acid linker connects the paired and homeodomains, contributing to minor groove interactions. The PST domain interacts with transcriptional coregulators, with its 40 C-terminal residues highly conserved and essential for linking HD to target sequences [1,23].

Pax6(5a) arises via inclusion of exon 5a, introducing a 14-residue insertion in the PD that modifies DNA-binding specificity [17,32]. Pax6 and Pax6(5a) are expressed in the diencephalon, telencephalon, and rhombencephalon, with canonical Pax6 mRNA levels 6–10 times higher than Pax6(5a) during early neurogenesis [34]. Pax6ΔPD, produced from an internal Pα promoter between exons 4 and 5 with an alternative start codon in exon 7, lacks the PD and has distinct functional properties [28,35,36]. The overexpression of Pax6(PD) induces a severe microphthalmic phenotype, which is caused by apoptotic cell death in the lens during embryonic development [36,37]. The PD is critical for regulating neurogenesis, proliferation, and proper brain development [27,38]. Pax6 expression is modulated by three promoters (P0, P1, Pα) and subject to both positive and negative autoregulation in vitro [39,40]. In the mouse optic vesicle, Pax6 overexpression suppresses neuroepithelial proliferation [41].

At the molecular level, Pax6 regulates the transcription of genes essential for embryonic development and organogenesis [13,45,46,47]. It is also noted that Pax6 is involved in maintaining multipotency in progenitor populations including retinal neurons, retinal pigment epithelium, iris [48,49,50], olfactory bulb neurons [51], and cortical/subcortical neurons [52,53]. Its multiple functional domains support roles in proliferation [54,55] and differentiation [29]. Pax6 competes with Pax4 for binding to insulin, glucagon and somatostatin promoters. Pax4 predominantly represses transcription, whereas Pax6 activates these genes [56].

In adults, Pax6 is involved in the formation of new neurons in the dentate gyrus and subventricular zone [57], specifying dopaminergic [58] and olfactory bulb neurons [59,60]. In the eye, it regulates iris cell proliferation and differentiation during lens regeneration [61]. In the pancreas, Pax6 governs cell-type specification and maintains endocrine function and glucose homeostasis [62].

Thus, Pax6 is an early universal regulator of morphogenesis, ensuring the correct formation of the eyes, neurons in the brain and pancreatic cells. Its ability to regulate gene expression at various levels makes it a key factor that coordinates complex developmental processes at the stage of embryogenesis, as well as an important factor in maintaining tissue homeostasis in adults.

### 2.2. Pax6 Expression and Pathways in CNS Development

During mammalian brain development, Pax6 exhibits tightly regulated spatiotemporal expression patterns. It is positioned upstream of multiple gene networks that govern brain regionalization, neuronal migration, and the formation of neural circuits [63,64]. At the neural plate stage, composed primarily of proliferating neuroepithelial cells, early Pax6 expression was detected at embryonic day 8 in mice and day 10 in rats. At this stage, Pax6 is present in the forebrain and spinal cord [65], within the germinal zone of the lateral ventricular wall, and in a limited population of neurons located in the marginal zone near the brain surface by embryonic day 10 [66]. Between embryonic days 10 and 12, the germinal layer expands, coinciding with the generation of a sufficient population of glutamatergic pyramidal neurons. In contrast, inhibitory γ-aminobutyric acid (GABA)-producing interneurons originate in the ventral telencephalon and subsequently migrate into the developing cortex [67,68]. Throughout this phase of neurogenesis, Pax6 expression is maintained in the germinal zone of the dorsal telencephalon and at the boundary between dorsal and ventral telencephalic regions [69]. Subsequently, the thickness of the germinal layer decreases and the ependymal layer is formed that expresses Pax6 [57]. The level of Pax6 expression maintains the balance between proliferation and differentiation of cortical neuronal progenitors [70].

Pax6 has been shown to bind and regulate a large number of promoters in neuronal progenitor cells. It plays a dual role by activating neuronal and ectodermal gene programs while simultaneously repressing mesodermal and endodermal genes, thereby establishing a unidirectional commitment to neuronal differentiation [45]. Pax6 plays a regulatory role at the earliest stages of cortical neurogenesis and is involved in the development of the forebrain. Studies using Pax6-null mouse models (Pax6-/-) have consistently shown severe cortical malformations, reduced neuronal numbers, and formation of an abnormally thin cortical plate [45,52,71,72,73,74,75]. Mechanistic analyses revealed that this phenotype results from premature depletion of the progenitor pool due to accelerated cell cycle exit [76]. In this paper [75], an analysis of the kinetics of the cell cycle was performed, and it was proven that loss of Pax6 leads to abnormally rapid cortical neurogenesis in vivo. In rodents with homozygous mutations of Pax6 (Pax6Sey/Sey), a decrease in the thickness of the forebrain and cortex was observed [72]. Patients with heterozygous Pax6 mutations show reduced frontal–parietal cortex thickness, a phenotype also observed in mice with small eyes [77].

Conversely, Pax6 overexpression has been shown to increase cortical thickness, particularly within the intermediate zone [55]. The researchers associate this phenomenon with the retention and proliferation of neurons in this region. It was found that the influence of Pax6 overexpression on cortical architecture and neuronal migration appears to be temporary and is thought to result from autoregulatory shifts in the ratio of Pax6 isoforms. The researchers do not exclude that Pax6 may modulate neuronal proliferation and migration through interactions with Wnt3 signaling.

In the development of the cortex researchers attach importance to the synergistic interaction of Pax6 with downstream target genes such as FABP7, Ngn2, p271, and Wnt7b1 [14,78,79]. In the lens, the neurogenesis is regulated by Pax6 signaling pathways involving Wnt-Dkk1, Sfrp1, and Sfrp2 [80]. In the rat embryo hindbrain, Pax6 has been shown to regulate expression of Cdh7 and Wnt5a [81]. It is known that expression of the neural stem cell marker Fabp7/BLBP gene encoding a protein of brain-type fatty acids is suppressed in the forebrain and hindbrain of homozygous Pax6 mutant rats [82].

Pax6 plays a key role in the development of the eye [82]. The absence of Pax6 causes neonatal lethality in mice as well as eye loss and cortical malformations [83]. Heterozygous mutations of Pax6 cause aniridia in humans and a small eye phenotype in mice [84,85]. In retinal development, which is considered an extension of the forebrain, Pax6 deficiency leads to premature neuronal differentiation associated with upregulation of Mash1 in Pax6-/- mutant mouse embryos [86]. In humans, Pax6 haploinsufficiency is associated with structural brain abnormalities, including pineal gland hypoplasia or absence, impaired olfactory bulb function, and unilateral polymicrogyria [87,88,89].

One of the key signaling pathways triggered by neurogenesis involving Pax6 is the Wnt/β-catenin pathway, a key regulator of stem cell maintenance and early neuroepithelial development. It has been established that β-catenin directly binds to the Pax6 promoter and activates its transcription. In β-catenin-deficient neural stem cells increased Pax6 expression partially restores impaired proliferation and neurogenesis. These findings identify Pax6 as a downstream effector of Wnt/β-catenin signaling and demonstrate that the β-catenin-Pax6 axis governs self-renewal and neurogenesis of radial glial cells during neocortical development [90].

In early neural tube development as well as in late retinal cell development Pax6 is involved in the fibroblast growth factor (FGF) signaling. Pax6 activation has been shown to occur during somitogenesis in the spinal cord. Presomitic mesoderm can inhibit Pax6 expression via the FGF signaling pathway. In presomitic mesoderm decreased Pax6 activation was observed in response to an FGF8 gradient. On the contrary, disruption of the FGF signal induces premature Pax6 expression in the neural plate. The authors conclude that the decrease in FGF8 activity marks the transition from early posterior neural epithelium to more advanced developmental stages [91]. In the spinal cord, Pax6 mediates FGF-dependent neuronal differentiation and is required for proper specification of ventral neuronal subtypes, as demonstrated in the small-eye mouse model (Sey/Sey). Pax6 controls the expansion of the dorsal neural tube [92,93] and changes in motor neuron specification [94]. Activation of Pax6 is regulated by signaling pathways that control neuronal differentiation. In the caudal neural tube, where stem cells are concentrated Pax6 is absent [95] as its activity is suppressed by fibroblast growth factor (FGF) to prevent premature neuronal differentiation [91,96]. Subsequent activation of Pax6 in the closing neural tube is driven by retinoic acid signaling in parallel with neural determinant expression [97,98]. The level of Pax6 protein is critical for cell cycle exit and initiation of neuronal differentiation. Once neural progenitors are recruited into the neural program, Pax6 expression must be downregulated to allow terminal differentiation. Loss-of-function studies in chick and mouse embryos show that Pax6 deficiency results in premature differentiation of neurons. Pax6 directly regulates the proneural gene Ngn2 in a dose-dependent manner, while high Ngn2 levels, in turn, suppress Pax6 expression, establishing a negative feedback loop [99]. FGF signaling also contributes to retinal epithelium development via Pax6-dependent pathways. According to the work [49], two principal targets of the Pax6/Mitf/Tfec network have been identified: Fgf15, which promotes retinal cell development, and Dkk3, which cooperates with FGF signaling to inhibit Wnt activity and support retinal pigment epithelium differentiation. Pax6 was shown to suppress expression of both Fgf15 and Dkk3. Pax6 has been reported to control cell adhesion properties, the neuronal polarity, the migration and the axon formation via a signaling pathway involving δ-catenin. The cell adhesion is impaired in the optic vesicle of embryos with a homophilic mutation of Pax6 [100]. Abnormalities in neuronal polarity in radial glia and granule cells of the cerebellum have been described in Pax6 mutants [101,102,103]. In this paper [104], δ-catenin was identified as a target of Pax6 in the CNS. Coexpression of the δ-catenin protein transcript with Pax6 was proven in the optic vesicle, the neocortex and the developing cerebellum. However, δ-catenin expression levels were significantly reduced in the CNS of Pax6 mutants. Pax6 directly binds conserved regulatory elements within the δ-catenin promoter, although additional transcription factors also contribute to δ-catenin regulation outside Pax6 expression domains.

In general, multiple converging signaling pathways regulate cytoskeletal dynamics during neurogenesis. Yamasaki et al. [102] proposed a link between Pax6 and the Rho GTPase and RhoA pathways in neuronal cytoskeletal dynamics. Inhibition of RhoA stimulates neurite outgrowth while mutation of Pax6 leads to neurite shortening. Pax6-dependent regulation of R-cadherin expression influences pioneer axon growth in the forebrain [9,103]. δ-Catenin serves as a molecular hub connecting cortactin and RhoA, thereby balancing neurite elongation and branching [105]. A stimulatory effect of neurite elongation under the action of δ-catenin, which at the same time suppresses the activity of RhoA during neurite branching was shown in experiments on cell cultures [105]. δ-catenin regulates cadherin turnover and links cortactin, RhoA, cadherins in the CNS [106].

Pax6 also participates in gliogenesis by regulating glutamate transporter expression. Glutamate signaling is critical in neurogenesis because it regulates astrocyte progenitor migration, and expression of the glutamate transporter GLT1 (EAAT2) at the astrocyte membrane ensures efficient glutamate clearance and limits excitotoxicity. In addition, GLT1 can function as a signal transduction molecule [107]. This work [108] presents evidence that astrocyte precursors migration to the cerebral cortex is regulated by glutamate signaling during postnatal development [108]. Pax6 directly induces GLT1 expression by binding to a conserved distal enhancer of the GLT1 gene [109]. There is evidence that the GLT1/EAAT2 transporter mediates glutamate uptake in vivo by astroglial cells. Reduced GLT1 expression leads to elevated extracellular glutamate levels and excitotoxic neuronal death [110]. Premature death with spontaneous seizures in mice with genetic deletion of GLT1 was demonstrated [111]. Co-culture of astrocytes with neurons induces GLT1 expression in astrocytes. This effect is mediated by both direct cell–cell contact and neuronal soluble factors [112], including cAMP [113] and epidermal growth factor [114]. Astrocytes lacking Pax6 display increased proliferative capacity and enhanced migratory potential [115], whereas exogenous Pax6 promotes GLT1 expression and astroglial maturation [109].

Thus, these findings identify Pax6 as a central transcriptional regulator acting at multiple stages of neurogenesis. Through direct and indirect interactions with diverse signaling pathways, Pax6 coordinates neuronal proliferation, differentiation, migration, and glial specification, making it an essential determinant of CNS development, including the retina, olfactory bulb, spinal cord, and multiple brain regions.

## 3. The Role of Pax6 in Glucose Metabolism and the Development of Metabolic Disorders

Pax6 plays a fundamental role in pancreatic development and the control of glucose homeostasis. Pax6 directs the differentiation of endocrine-committed progenitor cells during pancreatic development [116,117]. In the adult human pancreas, Pax6 is detected in all hormone-secreting cell types, indicating a broad function in fully differentiated endocrine cells [118]. Pax6 is capable of binding and activating the promoters of insulin and other β-cell genes in immortalized β-cell lines [117,119]. Deletion of Pax6 in adult mouse islet cells causes hyperglycemia with reduced expression of insulin, glucagon, and somatostatin and elevated ghrelin levels [62,120]. A decline in Pax6 expression has also been observed in β-cells of hyperglycemic and insulin-resistant mouse models. Pax6 is required to preserve appropriate β-cell gene expression profiles and to suppress alternative hormone genes as well as transcriptional regulators characteristic of other islet cell lineages [121]. The authors showed that Pax6 can function simultaneously as a transcriptional activator and repressor in differentiated β-cells by interacting with regulatory DNA elements located within promoters, enhancers, and silencers. Overall, Pax6 is essential for maintaining the distinct identity of individual islet cell types [62,117].

A metabolic reduction in Pax6 expression may contribute to the pathogenesis of diabetes. A moderate decrease in Pax6 expression has been reported in β-cells cultured under high glucose conditions [122] and in β-cells subjected to oxidative stress [123]. Human Pax6 haploinsufficiency alters β-cell function and leads to impaired glucose homeostasis, indicating that even partial modulation of Pax6 expression can have significant glycemic effects [124,125]. This work [121] proposed several potential mechanisms for the development of diabetes involving Pax6. Deletion of Pax6 results in a profound disruption of β-cell identity. Enhanced ghrelin secretion from mutant β-cells may further suppress insulin release from the remaining functional β-cells through mechanisms described in previous studies [126,127,128]. In addition, non-cell-autonomous expansion of somatostatin-producing cells may exacerbate the reduction in insulin secretion [129]. An increased α-cell population can further aggravate hyperglycemia and, when combined with Pax6 deficiency in β-cells, may lead to severe ketosis [121].

Pax6 deficiency has been shown to impair glucose metabolism in both murine models and humans [130]. One proposed mechanism involves Pax6 mutations causing deficiency of prohormone convertase 1/3 (PC1/3), which leads to defective proinsulin processing and subsequent disruption of glucose metabolism. However, evidence shows that patients carrying PC1/3 mutations and mice harboring the PC1/3 N222D mutation exhibit marked obesity [131,132]. Additional evidence suggests that Pax6sey/sey mice with homozygous Pax6 mutations display reduced levels of glucagon-like peptide-1 (GLP-1) in both the small and large intestine [133].

Pax6 is indispensable for normal α-cell function. It regulates transcription of the glucagon gene as well as key transcription factors, including cMaf, MafB, and NeuroD1/Beta2, which are critical for glucagon expression and α-cell differentiation [29]. Moreover, Pax6 controls the expression of genes encoding the processing enzyme PC2 and its molecular chaperone 7B2, which together mediate the conversion of proglucagon into mature glucagon [134]. These findings underscore the essential role of Pax6 in glucagon biosynthesis and α-cell lineage specification.

Gosmain et al. [119] identified multiple novel Pax6 target genes in pancreatic β-cells that are involved in β-cell differentiation and functional maintenance. In particular, Pax6 regulates the expression of insulin genes 1 and 2, PC2, PC1/3, Pdx1, cMaf, MafA, GK, GLUT2, Nkx6.1, GIPR, and GLP-1R in β-cells. Pax6 can bind to and activate the PC1/3, Nkx6.1, GK, GIPR, and GLP-1R genes via specific regulatory elements. Reduction in Pax6 expression in primary β-cells alters their function, primarily by impairing glucose-stimulated insulin secretion (GSIS) and also by reducing insulin biosynthesis and GLP1 action. Pax6 is therefore considered necessary for the maintenance of β-cell differentiation rather than for their initial formation [116]. By directly binding to and activating the promoters of key insulin-related genes, including MafA, Pdx1, and PC1/3, Pax6 plays a crucial role in insulin biosynthesis [130,135,136,137].

Dysfunction of Pax6-dependent regulation of pancreatic function and energy metabolism is recognized as major contributor to obesity [124,138], insulin resistance and elevated body mass index [139,140], and diabetes [140,141] in patients with aniridia. Oral glucose tolerance tests have shown impaired insulin secretion in all patients with the Pax6 gene mutation [124]. The study [142] presents a comprehensive metabolomic analysis of plasma markers in patients with congenital aniridia. Impaired oxidative stress regulation was identified as associated with an increase in the amount of glutathione synthesis metabolites, a decrease in taurine synthesis and plasmalogen levels, decreased lysophospholipid levels, and elevated concentrations of dicarboxylic and hydroxy fatty acids in plasma. Investigators note increasing insulin resistance in patients with aniridia which is associated with increased bile acid levels involved in lipid and carbohydrate metabolism. Taurolithcholate-3 sulfate increases by almost 2-fold were reported in patients with aniridia [142].

Metabolic defects in patients with aniridia also are accompanied by dysregulation of the superfamily of ligand-activated transcription factors, namely peroxisome proliferator-activated receptors (PPARs). PPARγ is known to interact with Pax6 in regulating glucagon gene transcription [143,144]. Pax6 is required for proper proglucagon gene expression in both pancreatic and intestinal tissues [145]. Thiazolidinediones suppress glucagon gene transcription by engaging the PPARγ-activated receptor and inhibiting Pax6 transcriptional activity [143]. PPARγ expression is reduced in limbal stem cells from aniridia patients [146] and in Pax6-edited cells [147]. In general, patients with aniridia are characterized by established metabolic disorders and changes in carbohydrate metabolism, such as insulin resistance, dyslipidemia, abdominal obesity, and an increased risk of developing type 2 diabetes mellitus.

Thus, within the metabolic context, accumulated evidence indicates that Pax6 plays a pivotal role in endocrine cell development [116,118], regulation of islet hormone gene expression (insulin, glucagon, and somatostatin) [117], and insulin secretion by β-cells [148]. These findings highlight its important role not only in adulthood but also in establishing β-cell identity in the mature organism [121,149].

## 4. Role of Pax6 in the Development of the Neurodegenerative Diseases

Pax6 serves a significant regulatory function not only in neurogenesis during embryonic neurogenesis but also in pathogenic signaling mechanisms linked to neurodegenerative diseases. Pax6 was established to control the expression of genes associated with neurodegeneration through multiple genetic cascades governing neuronal and glial growth, differentiation, and maturation. In the Pax6 gene knockdown state neurodegenerative markers BDNF, S100β, GFAP, p73α, NGN2, and p73δ were suppressed. Pax6 may be directly associated with p53- and TGFβ-dependent signaling pathways and indirectly involved in the modulation of redox-sensitive regulatory networks [150].

*Alzheimer’s disease*. Zhang et al. [151] identified Pax6 as a key molecular link between amyloid-β-induced signaling and tau protein hyperphosphorylation. Elevated Pax6 expression has been detected in the brains of APP transgenic mice as well as in patients diagnosed with Alzheimer’s disease. An increase in Pax6 and E2F1 protein levels, both of which regulate cell cycle progression and proliferative activity, was observed in neurons of the entorhinal cortex in individuals with mid-stage Alzheimer’s disease [152]. As Alzheimer’s disease progresses, gradual increases in Pax6 expression by 1.2 times in the initial stage and by 1.8 times in severe cases have shown in studies [153]. The molecular mechanism by which amyloid-β activates intracellular signaling pathways that stimulate the transcription factors c-Myb and Pax6, ultimately resulting in tau hyperphosphorylation, was described in [151]. The authors identified a potential target gene for Pax6. This is the kinase GSK3B (GSK-3b), which phosphorylates tau and contributes to neurofibrillary tangle formation. Amyloid-β-induced neurotoxicity promotes Pax6-dependent transactivation of GSK-3b. Elevated GSK-3β expression has been documented in multiple Alzheimer’s disease models and in postmortem brain tissue from affected individuals [154,155]. The molecular model proposed by the authors in this work [152] presents a pathway for activating expression of nuclear transcription factors through initiation of amyloid-β cell death signals CD1 and CDK4/6, resulting in hyperphosphorylation of the pRB factor. This starts the transcription factor E2F1, which subsequently induces transcription and translation of Pax6 and c-Myb genes within the nucleus. Additionally, activated E2F1 and c-Myb further enhance Pax6 transcription. Knockdown of Pax6 resulted in reduced total tau levels at both mRNA and protein stages. Pax6 silencing also significantly decreased the expression of tau-phosphorylating kinases, including Cdk5 and Mapk1. Therefore, the authors conclude that Pax6 regulates multiple downstream targets involved in Alzheimer’s disease pathogenesis and propose the E2F1/c-Myb/Pax6 signaling axis as a promising therapeutic target [152].

*Parkinson’s disease*. In the adult human brain, Pax6 may contribute to the mechanisms underlying Parkinson’s disease development. Increased survival of SH-SY5Y cells, accompanied by reduced apoptotic and oxidative stress markers following excessive expression of Pax6 was found in the study [156]. In a SH-SY5Y cell culture model, a relevant model of Parkinson’s disease, expression of the Pax6 gene was induced using tetracycline. Then, the cells were neurotoxicized with rotenone and 1 methyl-4 phenyl-1,2,3,6-tetrahydropyridine. It was found that Pax6 induction improved cell survival, reduced apoptosis and decreased the effect of oxidative damage on mitochondria by increasing cell resistance to programmed cell death. Researchers note that during Parkinson’s disease progression, the compact and reticular parts of the substantia nigra are differently vulnerable as the pathology progresses. The greatest degree of degeneration is observed in the compact part of the substantia nigra. The reticular part degenerates later as the disease progresses. Pax6 expression has been detected in the reticular region of the substantia nigra. It has been proven that Pax6 has a protective function in dopamine-producing cells [157]. No studies describing a direct interaction between Pax6 and alpha-synuclein were identified. However, there are indications that pathological alpha-synuclein itself is capable of regulating the cell cycle. A study conducted on PC-12 cells engineered to conditionally induce alpha-synuclein expression found increased proliferation rate, a higher proportion of cells in the S phase, elevated mitotic markers, and reduced expression of tumor suppressor regulators [158]. Scientists believe that early intracellular accumulation of alpha-synuclein in postmitotic neurons of the human midbrain is accompanied by changes in the expression of microRNA, leading to the activation of cell cycle genes [159].

*Amyotrophic lateral sclerosis*. Amyotrophic lateral sclerosis is a progressive neurodegenerative disorder characterized by muscle paralysis and degeneration of upper and lower motor neurons within the cortex, brainstem, and spinal cord [160]. The main contribution to the pathogenesis of this disease is made by immune imbalance, which develops as the disease progresses [161]. Progression of amyotrophic lateral sclerosis is regulated by a shift in the balance towards activation of proinflammatory immune factors [162]. Pax6 has been reported to participate in the regulation of inflammatory genes and oxidative stress responses in neuropathy. Studies have shown that hydrogen peroxide levels influence extracellular Pax6 expression in cultured cells derived from ocular (corneal) and pancreatic tissues [163]. Pax6 has an immunomodulatory function in the brain by activating microglia either through direct interaction with the ionized binding protein 1 (Iba1) or indirectly via inflammation associated with neurodegenerative processes [164]. Iba1 is a calcium binding protein involved in actin assembly, cellular migration and phagocytic activity in activated microglia [165]. The protein is triggered by the proinflammatory cytokine IFNγ and forms complexes with L-fimbrin and small RAC GTPases in activated microglial cells [166].

Thus, the regulatory involvement of Pax6 in neurodegenerative disease development is associated with age-related changes in the brain and its effect on the neuronal cell cycle, as well as intracellular signaling pathways. These pathways are realized either through phosphorylation, as seen in Alzheimer’s disease, or through mechanisms involving apoptosis and oxidative stress, as in Parkinson’s disease. In addition, in the aging brain, Pax6 performs an immunoregulatory function, which may be a decisive factor in changing the balance towards proinflammatory factors in the development of amyotrophic lateral sclerosis.

## 5. Molecular Mechanisms in the Development of Tumor Degeneration of Tissues with the Participation of Pax6

*Glioblastoma*. In gliomas, Pax6 predominantly functions as a tumor suppressor by limiting tumor growth [167]. Pax6 expression progressively decreases with increasing glioma malignancy grade [168,169]. Accordingly, Pax6 has been proposed as a prognostic marker for malignant astrocytic gliomas, as reduced Pax6 expression in anaplastic astrocytomas and glioblastomas correlates with unfavorable clinical outcomes [169]. Glioblastoma tissues exhibit markedly lower Pax6 levels compared with adjacent non-tumor brain tissue, whereas anaplastic astrocytomas show approximately three-fold higher Pax6 expression than glioblastomas [169]. Pax6 mutations in gliomas have not been identified, and the lower expression is thought to result from epigenetic alterations acquired during tumor progression [170].

MicroRNA-mediated regulation of Pax6 has been described in glioma cells [171]. It was demonstrated that miR-335 is significantly increased in gliomas. Inhibition of miR-335 increases Pax6 protein expression, whereas miR-335 overexpression promotes proliferation, colony formation, and invasion of U251 glioblastoma cells—effects that are reversed by Pax6 overexpression. Moreover, miR-335–induced invasion of U251 cells is mediated by modulation of matrix metalloproteinases MMP-2 and MMP-9 through Pax6 targeting. These findings support the conclusion that Pax6 is a direct target of miR-335 and exerts anti-oncogenic effects during glioma development.

It has been shown that the removal of Pax6 in U251 glioblastoma cells alters cell morphology, promotes their increased proliferation, migration and colony formation ability, and enhances their resistance to oxidative stress [172]. Glioblastoma cell proliferation and clonogenicity are significantly increased when miR-335 causes an approximately two-fold reduction in Pax6 expression. Other studies have shown that Pax6 overexpression in U251 cells reduces anchorage-independent growth in soft agar assays, although it does not significantly affect cell doubling time [168]. In vivo animal experiments have demonstrated suppression of cell growth by increasing their number in G1 and decreasing them in S phase with temporary overexpression of Pax6 via adenovirus, followed by induction of pronounced cell death. Repeated intracranial and subcutaneous implantation experiments in nude mice using Pax6-stable transfectants provided compelling evidence that Pax6 suppresses glioblastoma growth in vivo and significantly prolongs survival.

*Prostate cancer*. Prostate cancer is associated with abnormal neuroendocrine cell differentiation, with androgen signaling playing a central role in normal prostate development and function. Dysregulation of androgen receptor signaling is a key driver of prostate cancer progression [173]. A model to explain the origin of androgen-resistant neuroendocrine cells originating from a small stem cell niche has been proposed. The main strategy is androgen-protective therapy, which directs stem cells to differentiate into androgen-independent neuroendocrine cells [174]. Pax6 acts as a repressor of prostate cancer progression by inhibiting androgen receptor activity. Immunohistochemical analyses revealed higher Pax6 expression in normal epithelial cells adjacent to tumors than in cancer cells themselves [175]. Pax6 expression varies among prostate cancer cell lines and is lowest in the androgen-sensitive LNCaP line. Functional studies demonstrated that Pax6 suppresses androgen receptor transcriptional activity through direct protein–protein interaction.

Subsequent studies have demonstrated the coactivating function of the transcriptional coregulator SPBP in relation to androgen receptors. SPBP stimulates transcription of the probasin promoter and expression of the androgen receptor target gene PSA in LNCaP cells. Pax6 inhibits SPBP-mediated androgen receptor activation and interferes with SPBP recruitment to androgen receptor target promoters [176].

In addition to its tumor-suppressive role, Pax6 has been implicated in the transformation of the tumor from an androgen-dependent phenotype to an aggressive, therapy-resistant neuroendocrine prostate cancer (NEPC) phenotype [177]. NEPC is characterized by loss of androgen receptor expression and elevated neuronal markers, including synaptophysin, chromogranin A, and neuron-specific enolase. NEPC is highly aggressive and has not effective therapeutic treatments [178,179,180]. NEPC differentiation reflects a cell lineage switch to neuronal phenotypes that mimics embryonic neurogenesis. Given its role in neurogenesis, Pax6 is considered to be one of the molecular switches in signal transduction, reprogramming chromatin accessibility through the MET/STAT5a pathway, thereby increasing lineage plasticity. STAT5a suppresses the expression of the methyltransferases KMT5C and SMYD5, leading to reduced H4K20me3 levels, which compromises genomic stability. H4K20me3 downregulation alters the cell line phenotype and confers a transcriptional profile of neuroendocrine cancer [177].

*Breast tumor*. In breast cancer, Pax6 predominantly exhibits oncogenic properties. Specific microRNAs can suppress Pax6 expression, thereby inhibiting breast cancer cell proliferation and invasion [181,182]. Increased Pax6 expression is associated with poor prognosis [183], and Pax6 promoter methylation correlates with metastatic potential [184]. Jin et al. [181] demonstrated that Pax6 overexpression enhances breast cancer cell migration and metastasis by activating the TGF-β/SMAD signaling pathway and inducing epithelial–mesenchymal transition [181]. TGF-β initiates the interaction of TGF-β cytokine with TGF-βR II receptors which recruits TGF-β I for downstream signaling through SMAD [185,186]. MicroRNA-135b has been shown to inhibit TGF-β signaling [187]. Studies have demonstrated activation of the TGF-β/SMAD signaling in breast cancer metastasis [188,189,190]. Pax6 overexpression in MCF-7 and MDA-MB-231 cells reduces E-cadherin expression and increases levels of mesenchymal markers, including N-cadherin, fibronectin, and vimentin [181].

*Liver cancer*. In hepatocellular carcinoma, Pax6 functions as a tumor suppressor. Pax6 overexpression inhibits anchorage-independent growth and invasion of hepatocellular carcinoma cells through upregulation of E-cadherin and suppression of thrombospondin-1 [191]. Sequencing of liver cancer cells was performed, and it was found that the Pax6 gene promoter is highly methylated [192]. The regulatory role of Pax6 in liver carcinoma cell lines through natural killer cells has been proven. Pax6 also exerts anti-tumor effects via modulation of natural killer cell activity, suppressing the expression of metalloproteinase and reducing the secretion levels of the oncoproteins sMICA and sULBP2 [193].

*Pancreatic cancer*. Both the MET receptor tyrosine kinase and Pax6 are overexpressed in pancreatic cancer. The Pax6(5a) isoform is more abundant in pancreatic carcinoma cells than canonical Pax6, and both forms bind an enhancer element in the MET promoter, activating MET transcription [194]. The MET gene produces the c-MET protein, which is a receptor tyrosine kinase present on the surface of pancreatic cells. The ligand of this protein is the hepatocyte growth factor (HGF) produced by pancreatic stellate cells [195]. The HGF/c–MET interaction triggers uncontrolled proliferation, apoptosis evasion, and invasion via the MAP/ERK signaling pathway [196]. In addition, the phosphorylated region of c-MET is a docking site for the p85 subunit of phosphatylinositol 3-kinase (PI3K). This binding subsequently leads to a series of biochemical reactions that inhibit tumor suppressors and increase tumor cell survival through a signaling pathway [197].

*Lung cancer*. Pax6 acts as an oncogene, promoting tumor growth and the formation of its chemoresistance in lung cancer. In small-cell lung cancer, Pax6 enhances cell proliferation, cell cycle progression, and resistance to cisplatin- and etoposide-induced apoptosis by suppressing NOTCH signaling and upregulating Nanog expression [198]. Pax6 knockdown sensitizes tumor cells to chemotherapy. Pax6 also regulates stromal stem cell plasticity in small cell lung cancer through the NOTCH–Nanog axis, facilitating reprogramming into tumor cells [198]. Pax6 binds the promoters of pluripotency-associated genes such as Oct4 and Nanog, thereby modulating stemness programs [199].

In non-small cell lung cancer, high Pax6 expression correlates with reduced patient survival. Pax6 promotes migration, invasion, and EMT by activating ZEB2 transcription and downregulating E-cadherin via the PI3K/AKT pathway [200]. Additional studies indicate that Pax6 activates tumor suppressors such as PTEN and SFRP2, thereby inhibiting WNT signaling through repression of WNTB2 [201]. Pax6 also drives cell cycle progression by activating MAPK signaling and promoting cyclin D expression, facilitating the G1/S transition through ERK1/2 and p38 phosphorylation [202].

*Retinoblastoma*. Retinoblastoma is one of the most common tumors in newborns babies. Researchers attribute the leading role in the development of retinoblastoma to the inactivation of the Rb-1 suppressor gene [203]. Pax6 exhibits a dual role in retinoblastoma, functioning as either an oncogene or tumor suppressor depending on context. Pax6 overexpression regulates proliferation and apoptosis of retinoblastoma cells [204,205]. Lentiviral-mediated Pax6 overexpression enhances cell proliferation, suppresses caspase-3-dependent apoptosis, and alters p53-dependent cell cycle regulation [205]. It was found that suppression of the Pax6 gene using microRNA leads to inhibition of retinoblastoma cell growth and to apoptotic death of tumor cells. These changes were accompanied by increased regulation of P21 and P27 proteins, suppression of cdc2 protein [204]. Pax6 inhibition also reduces apoptosis through decreased expression of pro-apoptotic proteins such as Bax and p21 [204,206]. Pax6 expression is regulated by miR-365b-3p, which is downregulated in retinoblastoma tissues; restoration of miR-365b-3p suppresses Pax6 expression, induces cell cycle arrest, and promotes apoptosis [207].

Thus, Pax6 can function either as a tumor suppressor or an oncogenic factor depending on tissue context, molecular signaling environment, and interactions with the tumor microenvironment.

## 6. Conclusions

**Figure 2:**
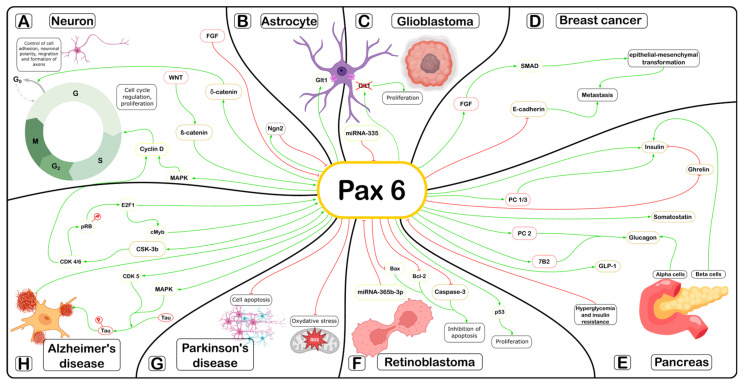
Participation of Pax6 in the implementation of intracellular signaling pathways: (**A**)—neurogenesis: green arrows show the signaling pathways of activation of Pax6 expression via the Wnt/β-catenin pathway during early neuroepithelial proliferation, as well as δ-catenin-dependent signaling during differentiation, adhesion and migration of neurons, red lines indicate inhibition of Pax6 activity by fibroblast growth factor (FGF) signaling during the differentiation of spinal cord neurons; (**B**)—gliogenesis: Pax6-dependent induction of the glutamate transporter GLT1 at the astrocyte plasma membrane during glial maturation and differentiation; (**C**)—glioblastoma: Pax6 acts as a downstream target of miR-335. Increased miR-335 expression suppresses Pax6, maintaining glioblastoma cells in a proliferative, undifferentiated state; (**D**)—breast tumor: green arrows indicate activation of the TGF-β/SMAD signaling pathway, promoting tumor cell proliferation and metastasis; (**E**)—metabolic disorders: Pax6 is essential for α- and β-cell function, controlling key genes for glucagon and insulin production. Its loss impairs hormone expression and secretion, leading to metabolic dysfunction; (**F**)—retinoblastoma: the promoter role of Pax6 in tumor development is demonstrated through suppression of apoptotic proteins. Tumor suppression is associated with Pax6 downregulation mediated by miR-365b-3p; (**G**)—Parkinson’s disease: Pax6 overexpression attenuates oxidative stress and neuronal apoptosis; (**H**)—Alzheimer’s disease: Pax6 activates the kinase GSK-3b, which in turn hyperphosphorylates the pRB factor via CD1 and CDK 4/6. pRB activates the transcription factor E2F1, which triggers transcription and translation of Pax6 and cMyb in the nucleus. Pax6 expression enhances the activity of Cdk5 and Mapk1 kinases, which phosphorylate the tau protein. Phosphorylated tau can, in turn, independently activate Pax6 expression in the nucleus.

The current level of scientific work demonstrates a whole spectrum of functions and signal regulatory pathways in which the Pax6 gene and its protein are involved. However, despite the diverse findings, the unifying role of Pax6 in the regulation of the cell cycle can be traced across both embryonic and adult stages. Various signaling pathways involving Pax6, as reviewed here, ultimately converge to regulate cell cycle duration in certain cell types. This process is critical during morphogenesis and in the development of pathophysiological conditions. Based on the available data, we propose a generalized scheme illustrating the involvement of Pax6 in various intracellular signaling pathways (Figure 2).

The most prominent Pax6-associated signaling pathway regulating the cell cycle in neuroepithelial cells during early embryogenesis is the Wnt/β-catenin pathway (Figure 2A). Binding of β-catenin to the Pax6 promoter, activates its transcription and ultimately triggers the MAP-kinase signaling pathway, which through the activation of cyclin D regulates cell cycle proliferation and the G1/S phase transition. During later stages of neurogenesis, Pax6 governs cell adhesion, neuronal polarity, and migration through δ-catenin-mediated signaling. Regulation of Pax6 expression is also essential for spinal cord development. Pax6 activation is important during somitogenesis. Later, when the presomitic mesoderm is formed, it inhibits Pax6 expression via the fibroblast growth factor (FGF8) signaling pathway.

The intracellular level of Pax6 protein is critical for terminating proliferation and initiating cell cycle exit during spinal cord neuronal differentiation. At this stage, neurogenin 2 (Ngn2) plays a decisive role in suppressing Pax6 expression and thereby triggering neuronal differentiation. Pax6 plays a significant role in glial differentiation during gliogenesis (Figure 2B). Pax6 carries out its regulatory role via glutamate signaling through the induction of expression of the glutamate transporter GLT1 at the astrocyte plasma membrane.

Pax6 can perform both a suppressive and oncogenic function during tumor development. In glioblastomas Pax6 acts as a tumor growth suppressor (Figure 2C). Its expression is regulated by microRNA (miR)-355. An increase in the concentration of miR-355 causes an approximately two-fold reduction in Pax6 expression, maintaining tumor cells in a highly proliferative and poorly differentiated state. In breast cancer, Pax6 acts as a promoter of the tumor process (Figure 2D). Activation of TGF-β/SMAD has been proven during breast tumor metastasis while Pax6 overexpression suppresses E-cadherin expression, thereby enhancing invasive potential of the cells.

During glioblastoma progression, cross-regulatory interactions have been identified between Pax6 isoforms and the miR-183-96-182 cluster. It has been demonstrated that canonical Pax6 is regulated by miR-182 and miR-96, whereas the Pax6(5a) isoform is preferentially targeted by miR-183 [208]. Molecular analysis in this study [208] revealed that Pax6 isoforms suppress glioma through two key changes: Pax6(5a) upregulates δ-catenin (CTNND2) expression, and both isoforms downregulate sphingosine kinase 1 (SPHK1). These combined effects reduce proliferation and enhance cell death in glioblastoma cells in vitro. Cultured astrocytes lacking Pax6 exhibit enhanced migratory potential, a characteristic reminiscent of high-grade glioma cells [115]. Both Pax6 isoforms significantly affect glioma cell viability and proliferation. Notably, Pax6(5a) exhibits a stronger tumor-suppressive capacity, suggesting that it may play a dominant role during early stages of glioma development [208].

Pax6 gene defects can affect ocular development and result in a wide range of clinical phenotypes, the most common of which is aniridia, a panophthalmologic disorder characterized by iris hypoplasia or absence, foveal hypoplasia, nystagmus, cataracts, and corneal keratopathy [209]. Additional ocular abnormalities include microphthalmia, anterior segment dysgenesis, and optic nerve defects [210]. Systemic manifestations may involve neurodevelopmental disorders such as attention deficit hyperactivity disorder and autism, speech impairments [211,212,213], and in some cases absent or malformed pineal and pituitary glands [88,89,214,215]. Pax6 dysfunction has also been linked to obesity and diabetes due to its essential role in pancreatic development and endocrine function [138,216].

The most frequent clinical manifestation of heterozygous Pax6 mutations is aniridia, largely a condition explained by Pax6 haploinsufficiency. The diversity of other observed phenotypes may be influenced by the specific molecular functions of different Pax6 isoforms. Structural differences within the PAI domain give rise to two principal Pax6 isoforms: isoform-a (Pax6-a) and isoform-b (Pax6-b). Their main distinction lies in the inclusion of an extra exon, exon 5a, within the PAI domain of Pax6-b. This insertion is thought to modify DNA-binding characteristics, including sequence selection or binding affinity [17,26,217]. Data from the literature indicate that these two isoforms regulate corneal epithelium-specific genes both differentially and synergistically, particularly keratin 12 (KRT12) and keratin 3 (KRT3) [218]. In the study [218], the upstream regulatory region of the KRT3 gene was shown to be a direct transcriptional target of Pax6-a [218]. Moreover, Pax6-a interacts with this region via the PAI domain, whereas Pax6-b binds through its RED domain. Both PAI and RED domains are required to induce expression of KRT3 and KRT12 [219].

Pax6 exhibits a dual function in retinoblastoma, acting both as an oncogenic promoter and as a tumor suppressor (Figure 2F). The promoter function of Pax6 is linked to inhibition of apoptosis through downregulation of caspase-3 and Bax. Pax6 also influences cell proliferation and cell cycle progression via the cdc25A/CDK2 and p53/p21/CDK1 signaling pathways. The suppressor function of Pax6 in the development of retinoblastoma is associated with the inhibitory effect of microRNA mir-365b-3p on Pax6, which causes cell cycle arrest and induction of tumor cell apoptosis.

During retinoblastoma development, the normal balance between the Pax6a and Pax6b isoforms is often disrupted. The predominance of the Pax6b isoform promotes retinal cell proliferation and migration [220]. However, the precise regulatory mechanisms and target genes of each isoform in this context remain incompletely understood. Canonical Pax6 shows higher expression during embryonic ocular development, where it is associated with differentiation and determination of cell fate. In contrast, the Pax6(5a) isoform becomes more prominent at later developmental stages and postnatally and is more closely linked to cell proliferation [221,222]. During embryonic development, studies in the mouse lens have demonstrated that canonical expression exceeds that of Pax6(5a) by approximately eight-fold. In adult ocular tissues, including the lens, retina, and cornea, the Pax6a/Pax6(5a) ratio shifts to approximately 1:1 [1,223]. A similar developmental shift has been observed in the chick embryo retina, where canonical Pax6 is highly expressed in early eye primordia and the lens placode, while Pax6(5a) expression gradually increases at later stages, especially in the lens and cornea [221]. Researchers suggest that these expression patterns may correlate with human phenotypes; for instance, mutations affecting the 5a-specific peptide insert in the paired domain (CTS) of Pax6(5a)—a key determinant of its DNA-binding specificity—are associated with isolated foveal hypoplasia [224,225]. Moreover, studies indicate that canonical Pax6 primarily induces KRT3 expression, whereas Pax6(5a) predominantly activates KRT12, with each isoform acting through its respective paired subdomain [218]. The precise ratio of these isoforms is critical for normal ocular development, and its dysregulation is linked to various eye pathologies, including retinoblastoma [218,220].

Pax6 plays a certain regulatory role in the development of neurodegenerative processes. In Parkinson’s disease, Pax6 improves cell survival in the dopamine formation by inhibiting oxidative stress and reducing neuronal apoptosis (Figure 2G). Pax6 acts as an important molecular intermediary linking amyloid-β-induced signaling to tau protein hyperphosphorylation (Figure 2H). At the same time, the main signaling pathways that are launched in this pathological process enhance the expression of Pax6. The main signaling pathway involved in Alzheimer’s disease progression includes E2F1, c-Myb, and Pax6.

Pax6 is a central transcriptional regulator of lineage specification and maintenance within pancreatic islets (Figure 2E). Pax6 is expressed in the pancreas beginning at embryonic day 9.5 and is restricted to cells already committed to the endocrine lineage, persisting postnatally in all islet cell types [116,226,227]. Distinct Pax6 domains perform specialized and independent roles in determining pancreatic endocrine cell fates. Analysis of Pax6-/- knockout mice indicates that Pax6 fulfills indispensable, non-redundant functions in glucagon-producing α-cells, which are completely absent in these mutants. Additional abnormalities include reduced β-cell mass and islet disorganization [118]. In contrast, mouse strains carrying inactivating Pax6 mutations (Pax6^SEY^, Pax6^SEY-NEU^), involving deletions of the homeodomain plus transactivation domain or solely the transactivation domain, respectively, display phenotypes distinct from full knockouts. In these mutants, α-cells are present but reduced in number [6,117,228], suggesting that α-cell differentiation largely proceeds normally except for impaired proglucagon gene expression [228]. Studies have shown that the paired domain of Pax6 is more critical for complete α-cell development than its transactivation domain [229]. Pax6 activates gene expression cooperatively with other transcription factors, particularly large Maf proteins [230,231]. In genes in cooperation with other transcription factors, particularly with large Maf proteins [230,231]. In Pax6^SEY-NEU^ mutant mice, activation of the proglucagon gene is achieved through recruitment of alternative transcription factors to Pax6 binding sites. However, loss of the paired domain function be cannot compensated for, indicating that this domain acts as the primary DNA-binding scaffold for Pax6-containing transcriptional complexes in pancreatic tissue [229]. Indeed, the proglucagon gene is a direct Pax6 target, with Pax6 binding to the G1 and G3 promoter elements mainly via its paired domain [231].

Evaluating Pax6 as a therapeutic target for pathologies like neurodegeneration and cancer is challenging due to its highly context-dependent role. Pax6 functions within intricate signaling networks that vary by disease and even by tissue, making its effects whether promoting or suppressing pathology difficult to predict. Consequently, while Pax6 may represent a viable target in specific pathophysiological conditions, the efficacy of such an approach will depend on the precise signaling substrate, pathway branching, and mediator redundancy. Future research priorities should therefore focus on elucidating the fine-tuned regulation of Pax6 isoforms and developing isoform-specific inhibitors tailored to the predominant form driving a given disease, such as aniridia, specific cancers, or metabolic disorders.

## Data Availability

No new data were created or analyzed in this study. Data sharing is not applicable to this article.

## Abbreviations

The following abbreviations are used in this manuscript:

AKT	Protein Kinase B
APP	Amyloid Precursor Protein
BDNF	Brain-Derived Neurotrophic Factor
BLBP	Brain Lipid-Binding Protein
cAMP	Cyclic Adenosine Monophosphate
CD1	Cluster of Differentiation 1
cdc2	Cell Division Cycle 2
Cdh7	Cadherin 7
CDK	Cyclin-Dependent Kinase
CMYD5	SET and MYND Domain-Containing Protein 5
CNS	Central Nervous System
CTNND2	Catenin Delta 2
DKK	Dickkopf-related protein
E2F1	E2F Transcription Factor 1
EAAT2	Excitatory Amino Acid Transporter 2
ERK	Extracellular Signal-Regulated Kinase
FABP7	Fatty Acid-Binding Protein 7
FGF	Fibroblast Growth Factor
GABA	Gamma-aminobutyric acid
GFAP	Glial Fibrillary Acidic Protein
GIPR	Gastric Inhibitory Polypeptide Receptor
GK	Glucokinase
GLP-1	Glucagon-like peptide-1
GLP-1R	Glucagon-Like Peptide-1 Receptor
GLT1	Glutamate Transporter 1
GLUT2	Glucose Transporter 2
GSIS	Glucose-stimulated insulin secretion
GSK3B	Glycogen Synthase Kinase 3 Beta
GTPase	Guanosine Triphosphatase
HGF	Hepatocyte Growth Factor
Iba1	Ionized Calcium-Binding Adapter Molecule 1
IFN	Interferon
KMT5c	Lysine Methyltransferase 5C
KRT3	Keratin 3
KRT12	Keratin 12
MafA	MAF BZIP Transcription Factor A
MET	Mesenchymal–Epithelial Transition Factor
Mitf	Microphthalmia-associated transcription factor
MMP	Matrix Metalloproteinase
c-Myb	Cellular Myb
NEPC	Neuroendocrine Prostate Cancer
NEUROD1	Neurogenic differentiation 1
Ngn2	Neurogenin 2
Nkx6.1	NK6 homeobox 1
Oct4	Octamer-Binding Transcription Factor 4
PAI	Paired-Amino-terminal Domain
PC	Proprotein Convertase
PD	Paired Domain
Pdx1	Pancreatic and Duodenal Homeobox 1
PI3K	Phosphatidylinositol 3-Kinase
PPAR	Peroxisome Proliferator-Activated Receptor
PST	Proline-Serine-Threonine-rich domain
RED	Repressor/Enhancer Domain
SFRP	Secreted Frizzled-Related Protein
sMICA	Soluble MHC Class I Chain-Related Protein A
SPBP	Stromelysin-1 PDGF-responsive element Binding Protein
SPHK1	Sphingosine kinase 1
STAT	Signal Transducer and Activator of Transcription
sULBP2	Soluble UL16-Binding Protein 2
Tfec	Transcription Factor EC
TGF	Transforming Growth Factor
Wnt	Wingless-related integration site
WNTB2	Wnt Family Member B2
ZEB2	Zinc Finger E-Box Binding Homeobox 2
7B2	Secretogranin V
